# *i*Bsu1103: a new genome-scale metabolic model of *Bacillus subtilis *based on SEED annotations

**DOI:** 10.1186/gb-2009-10-6-r69

**Published:** 2009-06-25

**Authors:** Christopher S Henry, Jenifer F Zinner, Matthew P Cohoon, Rick L Stevens

**Affiliations:** 1 Mathematics and Computer Science Department, Argonne National Laboratory, S. Cass Avenue, Argonne, IL 60439, USA; 2 Computation Institute, The University of Chicago, S. Ellis Avenue, Chicago, IL 60637, USA

## Abstract

**Background:**

*Bacillus subtilis *is an organism of interest because of its extensive industrial applications, its similarity to pathogenic organisms, and its role as the model organism for Gram-positive, sporulating bacteria. In this work, we introduce a new genome-scale metabolic model of *B. subtilis *168 called *i*Bsu1103. This new model is based on the annotated *B. subtilis *168 genome generated by the SEED, one of the most up-to-date and accurate annotations of *B. subtilis *168 available.

**Results:**

The *i*Bsu1103 model includes 1,437 reactions associated with 1,103 genes, making it the most complete model of *B. subtilis *available. The model also includes Gibbs free energy change (Δ_*r*_*G*'°) values for 1,403 (97%) of the model reactions estimated by using the group contribution method. These data were used with an improved reaction reversibility prediction method to identify 653 (45%) irreversible reactions in the model. The model was validated against an experimental dataset consisting of 1,500 distinct conditions and was optimized by using an improved model optimization method to increase model accuracy from 89.7% to 93.1%.

**Conclusions:**

Basing the *i*Bsu1103 model on the annotations generated by the SEED significantly improved the model completeness and accuracy compared with the most recent previously published model. The enhanced accuracy of the *i*Bsu1103 model also demonstrates the efficacy of the improved reaction directionality prediction method in accurately identifying irreversible reactions in the *B. subtilis *metabolism. The proposed improved model optimization methodology was also demonstrated to be effective in minimally adjusting model content to improve model accuracy.

## Abstract 2

A new and validated genome-scale metabolic model of Bacillus subtilis 168, iBsu1103, is presented that has significantly improved completeness and accuracy.

## Background

*Bacillus subtilis *is a naturally competent, Gram-positive, sporulating bacterium often used in industry as a producer of high-quality enzymes and proteins [1]. As the most thoroughly studied of Gram-positive and sporulating bacteria, *B. subtilis *serves as a model cell for understanding the Gram-positive cell wall and the process of sporulation. With its similarity to the pathogens *Bacillus anthracis *and *Staphylococcus aureus*, *B. subtilis *is also important as a platform for exploring novel medical treatments for these pathogens. Moreover, the natural competence of *B. subtilis *opens the way for simple and rapid genetic modification by homologous recombination [2].

For all these reasons, *B. subtilis *has been the subject of extensive experimental study. Every gene essential for growth on rich media is known [3]; 60 gene intervals covering 49% of the genes in the genome have been knocked out and the resulting phenotypes analyzed [4]; ^13^C experiments have been run to explore the cell response to mutations in the central carbon pathways [5]; and Biolog phenotyping experiments [6] have been performed to study the ability of *B. subtilis *to metabolize 271 different nutrient compounds [7].

As genome-scale experimental datasets begin to emerge for *B. subtilis*, genome-scale models of *B. subtilis *are required for the analysis and interpretation of these datasets. Genome-scale metabolic models may be used to rapidly and accurately predict the cellular response to gene knockout [8,9], media conditions [10], and environmental changes [11]. Recently, genome-scale models of the metabolism and regulation of *B. subtilis *have been published by Oh *et al. *[7] and Goelzer *et al. *[12], respectively. However, both of these models have drawbacks and limitations. While the Goelzer *et al. *model provides regulatory constraints for *B. subtilis *on a large scale, the metabolic portion of this model is limited to the central metabolic pathways of *B. subtilis*. As a result, this model captures fewer of the metabolic genes in *B. subtilis*, thereby restricting the ability of the model to predict the outcome of large-scale genetic modifications. While the Oh *et al. *metabolic model covers a larger portion of the metabolic pathways and genes in *B. subtilis*, many of the annotations that this model is based upon are out of date. Additionally, both models lack thermodynamic data for the reactions included in the models. Without these data, the directionality and reversibility of the reactions reported in these models is based entirely on databases of biochemistry such as the Kyoto Encyclopedia of Genes and Genomes (KEGG) [13,14]. Hence, directionality is often over-constrained, with a large number of reactions listed as irreversible (59% of the reactions in the Goelzer *et al. *model and 65% of the reactions in the Oh *et al. *model).

In this work, we introduce a new genome-scale model of *B. subtilis *based on the annotations generated by the SEED Project [15-17]. The SEED is an attractive source for genome annotations because it provides continuously updated annotations with a high level of accuracy, consistency, and completeness. The exceptional consistency and completeness of the SEED annotations are primarily a result of the subsystems-based strategy employed by the SEED, where each individual cellular subsystem (for example, glycolysis) is annotated and curated across many genomes simultaneously. This approach enables annotators to exploit comparative genomics approaches to rapidly and accurately propagate biological knowledge.

During the reconstruction process for the new model, we applied a group contribution method [18] to estimate the standard Gibbs free energy change of reaction (Δ_*r*_*G*'°) for each reaction included in the model. We then developed new extensions to an existing methodology [19-21] that uses these estimated Δ_*r*_*G*'° values along with the reaction stoichiometry to predict the reversibility and directionality of every reaction in the model. The Δ_*r*_*G*'° values reported for the reactions in the model may also be of use in applying numerous forms of thermodynamic analysis now emerging [22-24] to study the *B. subtilis *metabolism on a genome scale.

Once the reconstruction process was complete, we applied a significantly modified version of the GrowMatch algorithm developed by Kumar and Maranas [25] to fit our model to the available experimental data. In the GrowMatch methodology, an optimization problem is solved for each experimental condition that is incorrectly predicted by the original model, in order to identify the minimal number of reactions that must be added or removed from the model to correct the prediction. As a result, many equivalent solutions are generated for correcting each erroneous model prediction. We propose new solution reconciliation steps for the GrowMatch procedure to identify the optimal combination of GrowMatch solutions that results in an optimized model. We also propose significant alterations to the objective function of the GrowMatch optimization to improve the quality of the solutions generated by GrowMatch.

## Results

### Reconstruction of the Core *i*Bsu1103 model

We started the model reconstruction by obtaining the annotated *B. subtilis *168 genome from the SEED. This annotated genome consists of 2,691 distinct functional roles associated with 3,257 (79%) of the 4,114 genes identified in the *B. subtilis *168 chromosome. Of the functional roles included in the annotation, 50% are organized into SEED subsystems, each of which represents a single biological pathway such as histidine biosynthesis. The functional roles within subsystems are the focus of the cross-genome curation efforts performed by the SEED annotators, resulting in greater accuracy and consistency in the assignment of these functional roles to genes. Reactions were mapped to the functional roles in the *B. subtilis *168 genome based on three criteria: match of the Enzyme Commission numbers associated with the reaction and the functional role; match of the metabolic activities associated with the reaction and the functional role; and match of the substrates and products associated with the reaction and functional role [26]. In total, 1,263 distinct reactions were associated with 1,032 functional roles and 1,104 genes. Of these reactions, 88% were assigned to functional roles included in the highly curated SEED subsystems, giving us a high level of confidence in the annotations that form the basis of the *B. subtilis *model.

Often genes produce protein products that function cooperatively as a multi-enzyme complex to perform a single reaction. To accurately capture the dependency of such reactions on all the genes encoding components of the multi-enzyme complex, we grouped these genes together before mapping them to the reaction. We identified 111 such gene groups and mapped them to 199 distinct reactions in the *B. subtilis *model. Reactions were mapped to these gene groups instead of individual genes if: the functional roles assigned to the genes indicated that they formed a complex; multiple consecutive non-homologous genes were assigned to the same functional role; or the reaction represented the lumped functions of multiple functional roles associated with multiple genes.

The metabolism of *B. subtilis *is known to involve some metabolic functions that are not associated with any genes in the *B. subtilis *genome. During the reconstruction of the *B. subtilis *model, 71 such reactions were identified. While 19 of these reactions take place spontaneously, the genes associated with the remaining reactions are unknown. These reactions were added to the model as open problem reactions, indicating that the genes associated with these reactions have yet to be identified (Table S3 in Additional data files 1 and 2).

Data from Biolog phenotyping arrays were also used in reconstructing the *B. subtilis *model. The ability of *B. subtilis *to metabolize 153 carbon sources, 53 nitrogen sources, 47 phosphate sources, and 18 sulfate sources was tested by using Biolog phenotyping arrays [7]. Of the tested nutrients, *B. subtilis *was observed to be capable of metabolizing 95 carbon, 42 nitrogen, 45 phosphate, and 2 sulfate sources. Transport reactions are associated with genes in the *B. subtilis *168 genome for only 94 (51%) of these proven nutrients. Therefore, 73 open problem transport reactions were added to the model to allow for transport of the remaining Biolog nutrients that exist in our biochemistry database (Table S3 in Additional data files 1 and 2).

In total, the unoptimized SEED-based *B. subtilis *model consists of 1,405 reactions and 1,104 genes (Table 1). We call this model the Core *i*Bsu1103, where the *i *stands for *in silico*, the Bsu stands for *B. subtilis*, and the 1,103 stands for the number of genes captured by the model (one gene is lost during the model optimization process described later). In keeping with the modeling practices first proposed by Reed *et al. *[27], protons are properly balanced in the model by representing all model compounds and reactions in their charge-balanced and mass-balanced form in aqueous solution at neutral pH [28].

**Table 1:** Model content overview

Model	Core *i*Bsu1103	Optimized *i*Bsu1103	Oh *et al. *model
Number of genes	1,104 (26.8%)	1,103 (26.8%)	844
Total reactions	1,411	1,443	1,020
Reactions associated with genes	1,266 (89.7%)	1,263 (87.5%)	904 (88.6%)
Spontaneous reactions	20 (1.4%)	20 (1.4%)	2 (0.2%)
Open problem reactions	125 (8.9%)	160 (11.1%)	114 (11.2%)
Total compounds	1,144	1,145	988

### Construction of a biomass objective function

In order to use the reconstructed *i*Bsu1103 model to predict cellular response to media conditions and gene knockout, a biomass objective function (BOF) was constructed. This BOF was based primarily on the BOF developed for the Oh *et al. *genome-scale model of *B. subtilis *[7]. The 61 small molecules that make up the Oh *et al. *BOF can be divided into seven categories representing the fundamental building blocks of biomass: DNA, RNA, lipids, lipoteichoic acid, cell wall, protein, and cofactors and ions. In the Oh *et al. *BOF, all of these components are lumped together as reactants in a single biomass synthesis reaction, which is not associated with any genes involved in macromolecule biosynthesis. In the *i*Bsu1103 model, we decomposed biomass production into seven synthesis reactions: DNA synthesis; RNA synthesis; protein synthesis; lipid content; lipoteichoic acid synthesis; cell wall synthesis; and biomass synthesis. These abstract species produced by these seven synthesis reactions are subsequently consumed as reactants along with 22 cofactors and ionic species in the biomass synthesis reaction. This process reduces the complexity of the biomass synthesis reaction and makes the reason for the inclusion of each species in the reaction more transparent. Additionally, this allows the macromolecule synthesis reactions to be mapped to macromolecule biosynthesis genes in *B. subtilis*. For example, genes responsible for encoding components of the ribosome and genes responsible for tRNA loading reactions were all assigned together as a complex associated with the protein synthesis reaction.

Some of the species acting as biomass precursor compounds in the Oh *et al. *BOF were also altered in the adaptation of the BOF to the *i*Bsu1103 model. In the Oh *et al. *model, the BOF involves 11 lumped lipid and teichoic acid species, which represent the averaged combination of numerous lipid compounds with varying carbon chain lengths. In the development of the fatty acid and cell wall biosynthesis pathways for the *i*Bsu1103 model, we represented every distinct fatty acid and teichoic acid species explicitly rather than using lumped reactions and compounds. As a result, lumped species that serve as biomass components in the Oh *et al. *model were replaced by 99 explicit species in the *i*Bsu1103 BOF. Of these species, 63 serve as reactants in the lipid content reaction, while the remaining species serve as reactants in the teichoic acid synthesis reaction.

Two new biomass precursor compounds were added to the biomass synthesis reaction of the *i*Bsu1103 model to improve the accuracy of the gene essentiality predictions: coenzyme A (CoA) and acyl-carrier-protein (ACP). Both of these species are used extensively as carrier compounds in the metabolism of *B. subtilis*, making the continuous production of these compounds essential. The biosynthesis pathways for both compounds already existed in the *i*Bsu1103, and two of the steps in these pathways are associated with essential genes in *B. subtilis*: ytaG (peg.2909) and acpS (peg.462). If these species are not included in the BOF, these pathways become non-functional, and the essential genes associated with these pathways are incorrectly predicted to be nonessential.

The coefficients in the Oh *et al. *BOF are derived from numerous analyses of the chemical content of *B. subtilis *biomass [29-33]. We similarly derived the coefficients for the *i*Bsu1103 model from these sources. While no data were available on the percentage of *B. subtilis *biomass represented by our two additional biomass components CoA and ACP, we assume these components to be 0.5% of the net mass of cofactors and ions represented in the BOF.

### Results of automated assignment of reaction reversibility

**Figure 1:**
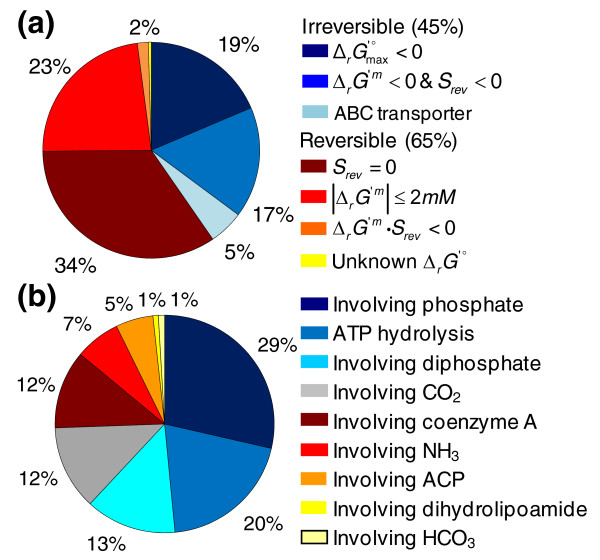
Distribution of reactions conforming to reversibility rules. **(a) **The distribution of reactions in the *i*Bsu1103 model conforming to every possible state in the proposed set of rules for assigning reaction directionality and reversibility is shown. This distribution indicates that most of the irreversible reactions in the model were determined to be irreversible because the Δ_*r*_*G*^' ^_*max *_value calculated for the reaction was negative. **(b) **The distribution of reactions in the *i*Bsu1103 model involving the compounds used in the reversibility score calculation is also shown. These compounds are prevalent in the reactions of the *i*Bsu1103 model, with 64% of the reactions in the model involving at least one of these compounds.

The group contribution method [18] was used to estimate standard Gibbs free energies of formation (Δ_*f*_*G*'°) for 948 (83.3%) of the metabolites and Δ_*r*_*G*'° for 1,372 (97.4%) of the reactions in the unoptimized *i*Bsu1103 model. Estimated Δ_*r*_*G*'° values were used in combination with a set of heuristic rules (see Materials and methods) to predict the reversibility and directionality of each reaction in the model under physiological conditions (Figure 1). Based on these reversibility rules, 635 (45%) of the reactions in the model were found to be irreversible. However, when the directionality of the irreversible reactions was set according to our reversibility criteria, the model no longer predicted growth on LB or glucose-minimal media. This result indicates that the direction of flux required for growth under these media conditions contradicted the predicted directionality for some of the irreversible reactions in the model. Six reactions were identified in the model that met these criteria (Table 2). In every case, these reactions were irreversible in the reverse direction because the minimum Gibbs free energy change () of each reaction was greater than zero. However, all of these reactions involve uncommon molecular substructures for which few experimental thermodynamic data are available [18]. Thus, in combination with the strong experimental evidence for the activity of these reactions in the direction shown in Table 2, we assumed that the Δ_*r*_*G*'° values of these reactions were overestimated by the group contribution method and that these reactions are, in fact, reversible.

**Table 2:** Reactions required to violate the automated reversibility rules

Reaction name	Equation	Δ_*r*_*G*^'*m *^(kcal/mol)
CMP-lyase	2-p-4-CDP-2-m-eryth => CMP + 2-m-eryth-2-4-cyclodiphosphate	22.7
Dihydroneopterin aldolase	Dihydroneopterin => Glycolaldehyde + 2-Amino-4-hydroxy-6-hydroxymethyl-7,8-dihydropteridine	10.7
Tetrahydrodipicolinate acetyltransferase	H_2_O + Acetyl-CoA + Tetrahydrodipicolinate => CoA + L-2-acetamido-6-oxopimelate	11.4
Dihydroorotase	H^+ ^+ N-carbamoyl-L-aspartate => H_2_O + L-dihydroorotate	5.3
Phosphoribosyl aminoimidazole synthase	ATP + 5'-Phosphoribosylformylglycinamidine => ADP + Phosphate + H^+ ^+ Aminoimidazole ribotide	16.6
Sulfate adenylyltransferase	ATP + Sulfate + H^+ ^=> Diphosphate + Adenylyl sulfate	12.6

### Results of the model optimization procedure

The unoptimized model was validated against a dataset consisting of 1,500 distinct experimental conditions, including gene essentiality data [3], Biolog phenotyping data [7], and gene interval knockout data [4] (Table 3). Initially, 85 errors arose in the gene essentiality predictions, including 58 false positive*s *(an essential gene being predicted to be nonessential) and 27 false negative*s *(a nonessential gene being predicted to be essential). The annotations of all erroneously predicted essential and nonessential genes were manually reviewed to identify cases where the prediction error was a result of an incorrect gene annotation. Of the essential genes that were predicted to be nonessential, 30 were mapped to essential metabolic functions in the model. However, these essential genes all had homologs in the *B. subtilis *genome that were mapped to the same essential metabolic functions (Table S4 in Additional data files 1 and 2). Three explanations exist for the apparent inactivity of these gene homologs: they are similar to the essential genes but actually perform a different function; they are nonfunctional homologs; or the regulatory network in the cell deactivates these genes, making them incapable of taking over the functions of the essential genes when they are knocked out. In order to correct the essentiality predictions in the model, these 30 homologous genes were disassociated from the essential metabolic functions.

**Table 3:** Accuracy of model predictions before and after optimization

Data type	Experimental data	Core *i*Bsu1103 (correct/total)	Fit *i*Bsu1103 (correct/total)	Oh *et al. *model (correct/total)
Biolog media with nonzero growth	184 [7]	107/184 (58.2%)	137/184 (74.5%)	122/184 (66.3%)
Biolog media with zero growth	87 [7]	80/87 (92%)	81/87 (93.1%)	79/87 (90.8%)
Essential genes in LB media	271 [3]	187/215 (87%)	192/215 (89.3%)	63/91 (69.2%)
Nonessential genes in LB media	3,841 [3]	862/889 (97%)	872/888 (98.2%)	657/675 (97.3%)
Nonessential intervals in LB media	63 [4]	55/63 (87.3%)	58/63 (92.1%)	58/63 (92.1%)
Nonessential intervals in minimal media	54 [4]	48/54 (88.9%)	49/54 (90.7%)	50/54 (92.6%)
Essential gene intervals in minimal media	9 [4]	5/9 (55.6%)	5/9 (55.6%)	6/9 (66.7%)
Overall accuracy	4,452	1,344/1,501 (89.5%)	1,398/1,500 (93.2%)	1,035/1,163 (89.0%)

LB, Luria-Bertani.

**Figure 2:**
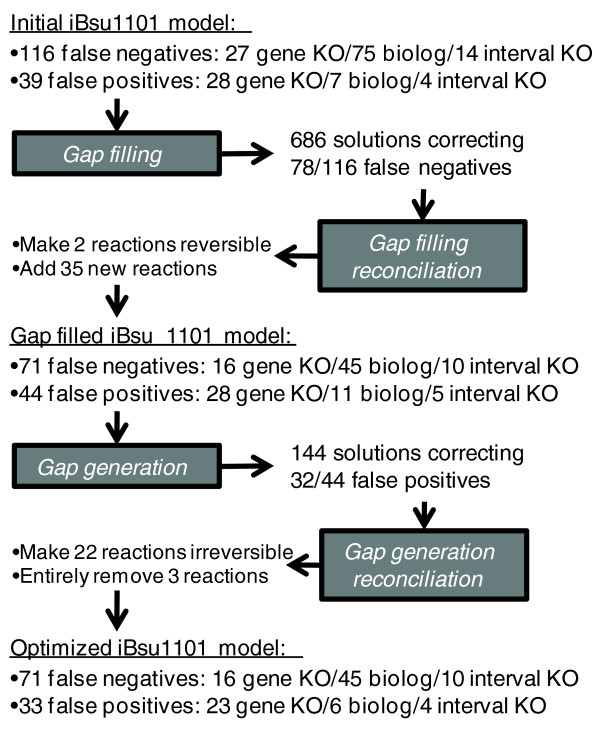
Model optimization procedure results. The results are shown from the application of each step of the model optimization procedure to fit the *i*Bsu1103 model to the 1,500 available experimental data-points. KO, knock out.

We then applied our modified GrowMatch model optimization procedure (see Materials and methods) in an attempt to fix the 116 remaining false negative predictions and 39 remaining false positive predictions (Figure 2). First, the gap filling algorithm was applied to identify existing irreversible reactions that could be made reversible or new reactions that could be added to correct each false negative prediction. This step produced 686 solutions correcting 78 of the false negative predictions. The gap filling reconciliation algorithm was used to combine the gap filling solutions into a single solution that corrected 45 false negative predictions and introduced five new false positive predictions. Next, the gap generation algorithm was applied to identify reactions that could be removed or made irreversible to correct each false positive prediction. The gap generation algorithm produced 144 solutions correcting 32 of the false positive predictions. The gap generation reconciliation algorithm combined these solutions into a single solution that corrected 11 false positive predictions without introducing any new false negative predictions. Overall, two irreversible reactions were made reversible, 35 new reactions were added to the model, 21 reversible reactions were made irreversible, and 3 reactions were removed entirely from the model (Table S5 in Additional data files 1 and 2). As a result of these changes, the model accuracy increased from 89.7% to 93.1%.

### Model overview

The final optimized version of the *i*Bsu1103 model consists of 1,437 reactions, 1,138 metabolites, and 1,103 genes (Table 1). Based on the reversibility rules and the estimated thermodynamic data, 653 (45.0%) of the model reactions were determined to be irreversible. All data relevant to the model are provided in the Additional data files, including metabolite structures (Additional data file 3), metabolite data (Table S1 in Additional data files 1 and 2), reaction data (Table S2 in Additional data files 1 and 2), estimated thermodynamic data (Table S2 in Additional data files 1 and 2), model stoichiometry in SBML format (Additional data file 4), and mappings of model compound and reaction IDs to IDs in the KEGG and other genome-scale models (Tables S1 and S2 in Additional data files 1 and 2).

**Figure 3:**
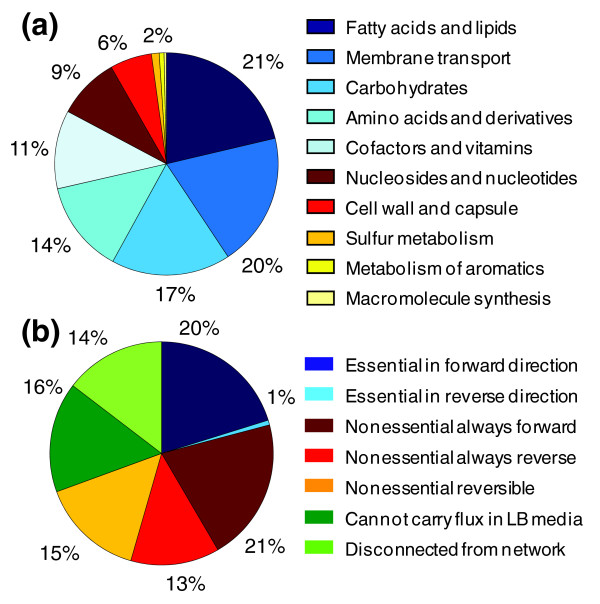
Classification of model reactions by function and behavior. **(a) **Reactions in the optimized *i*Bsu1103 model are categorized into ten regions of the *B. subtilis *metabolism. Regions of metabolism involving a diverse set of substrates typically involve the greatest number of reactions. **(b) **The *i*Bsu1103 reactions were also categorized according to their essentiality during minimal growth on Luria-Bertani (LB) media.

The reactions included in the optimized model were categorized into ten regions of *B. subtilis *metabolism (Figure 3a; Table S2 in Additional data files 1 and 2). The largest category of model reactions is 'fatty acid and lipid biosynthesis'. This is due to the explicit representation of the biosynthesis of every significant lipid species observed in *B. subtilis *biomass as opposed to the lumped reactions used in other models. The explicit representation of these pathways has numerous advantages: Δ_*f*_*G*'° and Δ_*r*_*G*'° may be estimated for every species and reaction; every species has a distinct structure, mass, and formula; and the stoichiometric coefficients in the reactions better reflect the actually biochemistry taking place. The other most significantly represented categories of model reactions are carbohydrate metabolism, amino acid biosynthesis and metabolism, and membrane transport. These categories are expected to be well represented because they represent pathways in the cell that deal with a highly diverse set of substrates: 20 amino acids, more than 95 metabolized carbon sources, and 244 transportable compounds.

Reactions in the model were also categorized according to their behavior during growth on Luria-Bertani (LB) media (Figure 3b; Table S2 in Additional data files 1 and 2). Of the model reactions, 300 (21%) were essential for minimal growth on LB media. These are the reactions fulfilling essential metabolic functions for *B. subtilis *where no other pathways exist, and they form an always-active core of the *B. subtilis *metabolism. Another 697 (49%) of the model reactions were nonessential but capable of carrying flux during growth on LB media. While these reactions are not individually essential, growth is lost if all of these reactions are simultaneously knocked out. The reason is that some of these reactions represent competing pathways for performing an essential metabolic function. Another 229 (16%) of the reactions cannot carry flux during growth on LB media. These reactions are on the periphery of the *B. subtilis *metabolism involved in the transport and catabolism of metabolites not included in our *in silico *representation of LB media. Moreover, 210 (14%) of the model reactions are disconnected from the network, indicating that these reactions either lead up to or are exclusively derived from a dead end in the metabolic network. Presence of these reactions indicates miss-annotation or overly generic annotation of the gene associated with the reaction, or a gap in the metabolic network. Thus, these reactions represent areas of the metabolic chemistry where more experimental study and curation of annotations must occur.

### Comparison with previously published models of *B. subtilis*

**Figure 4:**
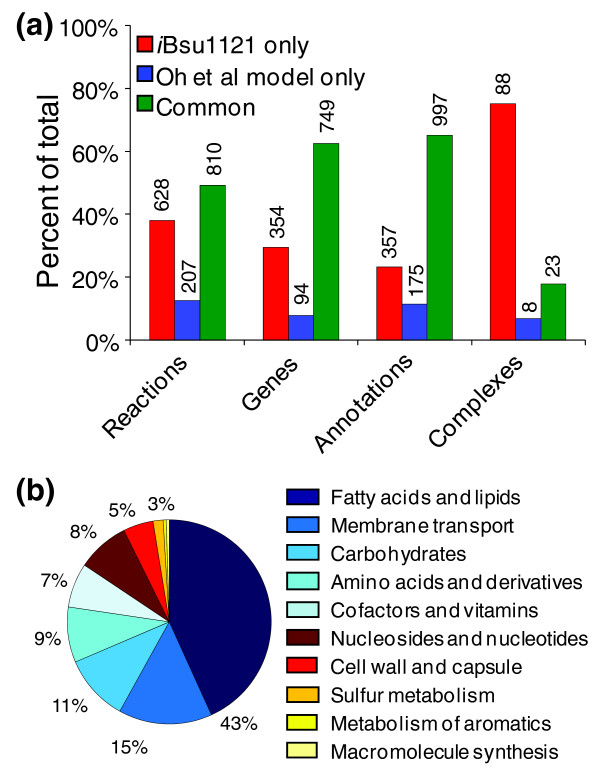
Comparison of *i*Bsu1103 model to the Oh *et al. *model. **(a) **A detailed comparison of the *i*Bsu1103 model and the Oh *et al. *model was performed to determine overlap of reactions, genes, annotations, and gene complexes between the two models. In the annotation comparison, only annotations involving the 818 overlapping reactions in the two models were compared; and each annotation consisted of a single reaction paired with a single gene. If two genes were mapped to a single reaction, this was treated as two separate annotations in this comparison. **(b) **The distribution of the 628 reactions that are exclusive to the *i*Bsu1103 model among the metabolic pathways of the cell. Almost half of the exclusive reactions in the *i*Bsu1103 model are involved in the Fatty Acids and Lipids pathway due to the unlumping of these reaction pathways in the *i*Bsu1103 model.

We performed a detailed comparison of the Oh *et al. *and *i*Bsu1103 models to identify differences in content and elucidate the conflicts in the functional annotation of genes (Table 1). Our comparison encompassed the reactions involved in the models, the genes involved in the models, the mappings between genes and reactions in the models, and the gene complexes captured by the models (Figure 4). Our comparison revealed significant overlap in the content of the two models. Of the 1,020 total reactions in the Oh *et al. *model, 810 (79%) were also contained in the *i*Bsu1103 model. The remaining 210 Oh *et al. *reactions were excluded from the *i*Bsu1103 model primarily because of a disagreement between the Oh *et al. *and SEED annotations or because they were lumped reactions that were represented in un-lumped form in the *i*Bsu1103 model (Table S6 in Additional data files 1 and 2).

Significant agreement was also found in the mapping of genes to reactions in the Oh *et al. *and *i*Bsu1103 models. Of the 1,550 distinct gene-reaction mappings that involved the 810 reactions found in both models, 997 (64%) were identical. Of the 357 mappings that were exclusive to the *i*Bsu1103 model, 20 involved reactions that were included in the Oh *et al. *model without any gene association. The remaining 337 exclusive *i*Bsu1103 mappings involved paralogs or gene complexes not captured in the Oh *et al. *annotation. The 175 mappings exclusive to the Oh *et al. *model all represent conflicts between the functional annotations in the Oh *et al. *model and the functional annotations generated by the SEED. Although some of these Oh *et al. *exclusive mappings involved eight reactions with no associated gene in the *i*Bsu1103 model, these mappings were rejected because they conflicted with the SEED annotation.

In addition to containing most of the reaction and annotation content of the Oh *et al. *model, the *i*Bsu1103 model also includes 628 reactions and 354 genes that are not in the Oh *et al. *model (Figure 4; Table S2 in Additional data files 1 and 2). Of the additional reactions in the *i*Bsu1103 model, 173 are associated with the 354 genes that are exclusive to the *i*Bsu1103 model. These additional reactions are a direct result of the improved coverage of the *B. subtilis *genome by the SEED functional annotation. The remaining 455 reactions that are exclusive to the *i*Bsu1103 model take part in a variety of functional categories spread throughout the *B. subtilis *metabolism, although nearly half of these reactions participate in the fatty acid and lipid biosynthesis (Figure 4b). These reactions are primarily a result of the replacement of lumped fatty acid and lipid reactions in the Oh *et al. *model with unlumped reactions in the *i*Bsu1103 model.

A comparison of the gene complexes encoded in both model reveals little overlap in this portion of the models. Of the 111 distinct gene complexes encoded in the *i*Bsu1103 model, only 21 overlapped with the Oh *et al. *model, whereas the Oh *et al. *model contained only 8 gene complexes not encoded in the *i*Bsu1103 model (Figure 3). This indicates a significantly more complete handling of complexes in the *i*Bsu1103 model.

All of the additional content in the *i*Bsu1103 model translates into a significant improvement in the accuracy of the gene knockout predictions, the Biolog media growth predictions, and the gene interval knockout predictions (Table 3). Even before optimization, the *i*Bsu1103 model is 0.7% more accurate than the Oh *et al. *model. After optimization, the *i*Bsu1103 model is 4.1% more accurate. In addition to the improvement in accuracy, the improved coverage of the genome by the *i*Bsu1103 model also allows for the simulation of 337 additional experimental conditions by the model.

We note that while the annotations used in the *i*Bsu1103 model were derived primarily from the SEED, the Oh *et al. *model proved invaluable in reconstructing the *i*Bsu1103 model. The work of Oh *et al. *was the source of Biolog phenotyping data and analysis; and the Oh *et al. *model itself was a valuable source of reaction stoichiometry, metabolite descriptions, and data on biomass composition, all of which were used in the reconstruction of the *i*Bsu1103 model.

## Conclusions

As one of the first genome-scale metabolic models constructed based on an annotated genome from the SEED framework, the *i*Bsu1103 model demonstrates the exceptional completeness and accuracy of the annotations generated by the SEED. The *i*Bsu1103 model covers 259 more genes than the Oh *et al. *model; it can simulate 337 more experimental conditions; and it simulates conditions with greater accuracy. In fact, of the seven new assignments of functions to genes proposed in the Oh *et al. *work based on manual gene orthology searches, two were already completely captured by the SEED annotation for *B. subtilis *168 prior to the publication of the Oh *et al. *manuscript. Another two of these proposed annotations were partially captured by the SEED annotation.

In this work we also demonstrate new extended reversibility criteria for consistently and automatically assigning directionality to the biochemical reactions in genome-scale metabolic models. The extended criteria enabled us to identify 306 additional irreversible reactions that are missed when using existing methodologies alone [19-21]. However, we also found that even with the extended criteria, the predicted reversibility was not correct for every reaction in the model. In order for model predictions to fit available experimental observations, the predicted reversibility had to be adjusted for 29 (2%) of the model reactions. Some possible explanations for these exceptions to the reversibility criteria include: the estimated Δ_*r*_*G*'° may be too high or too low; the reactant or product concentrations may be tightly regulated to levels that prohibit reactions from functioning in certain directions; or the reactions involve additional/alternative cofactors not accounted for in current reversibility calculations. These exceptions to the reversibility rules emphasize the importance of using a model correction method to adjust predicted reversibility based on experimental data. While these rules were very effective with the *i*Bsu1103 model, they still need to be validated with a wider set of organisms and models. The extended version of GrowMatch presented in this work was also demonstrated to be a highly effective means of identifying and correcting potential errors in the metabolic network that cause errors in model predictions. This method is driven entirely by the available experimental data, requiring manual input only in selecting the best of the equivalent solutions generated by the solution reconciliation steps of the method. The reconciliation steps we introduced to the GrowMatch method also proved to be effective for identifying the minimal changes to the model required to produce the optimal fit to the available experimental data. The reconciliation reduced 830 distinct solutions involving hundreds of changes to the model to a single solution that combined 62 model modifications to fix 51 (33%) of the 155 incorrect model predictions.

Overall, we demonstrate the *i*Bsu1103 model to be the most complete and accurate model of *B. subtilis *published to date. The identification and encoding of gene complexes, the removal of lumped reactions and compounds, and the refinements of the biomass objective function make this model especially applicable to thermodynamic analysis and gene knockout prediction. This model will be a valuable tool in the ongoing efforts to genetically engineer a minimal strain of *B. subtilis *for numerous engineering applications [2,4]. The thermodynamic data published with this model will be invaluable in the application of the model to numerous emerging forms of thermodynamic analysis [22-24]. Additionally, the new extensions that we have proposed for methods of automatically predicting reaction reversibility and automatically correcting model errors are valuable steps towards the goal of automating the genome-scale model reconstruction process [34,35].

## Materials and methods

### Validation of the *B. subtilis *model using flux balance analysis

Flux balance analysis (FBA) was used to simulate all experimental conditions to validate the *i*Bsu1103 model. FBA defines the limits on the metabolic capabilities of a model organism under steady-state flux conditions by constraining the net production rate of every metabolite in the system to zero [36-39]. This quasi-steady-state constraint on the metabolic fluxes is described mathematically in Equation 1:

(1)

In Equation 1, *N *is the *m *× *r *matrix of the stoichiometric coefficients for the *r *reactions and *m *metabolites in the model, and *v *is the *r *× 1 vector of the steady-state fluxes through the *r *reactions in the model. Bounds are placed on the reaction fluxes depending on the reversibility of the reactions:

(2)

(3)

- (CDW = cell dry weight). When simulating a gene knockout, the bounds on the flux through all reactions associated exclusively with the gene being knocked out (or associated exclusively with a protein complex partially encoded by the gene being knocked out) were reset to zero. When simulating media conditions, only nutrients present in the media were allowed to have a net uptake by the cell. All other transportable nutrients were allowed only to be excreted by the cell. Details on conditions for all FBA simulations performed are provided in Table S8 in Additional data files 1 and 2.

### Prediction of reaction reversibility based on thermodynamics

The reversibility and directionality of the reactions in the *i*Bsu1103 model were determined by using a combination of thermodynamic analysis and a set of heuristic rules based on knowledge of metabolism and biochemistry. In the thermodynamic analysis of the model reactions, Δ_*r*_*G*'° was estimated for each reaction in the model by using the group contribution method [40-42]. The estimated Δ_*r*_*G*'° values were then used to determine the minimum and maximum possible values for the absolute Gibbs free energy change of reaction (Δ_*r*_*G*^'^) using Equations 4 and 5, respectively:

(4)

(5)

In these equations, *x*_*min *_is the minimal metabolite activity, assumed to be 0.01 mM; *x*_*max *_is the maximum metabolite activity, assumed to be 20 mM; *R *is the universal gas constant; *T *is the temperature; *n*_*i *_is the stoichiometric coefficient for species *i *in the reaction; *U*_*r *_is the uncertainty in the estimated Δ_*r*_*G*'°; and Δ*G*_*Transport *_is the energy involved in transport of ions across the cell membrane. Any reaction with a negative maximum Gibbs free energy change of reaction () was assumed to be irreversible in the forward direction, and any reaction with a positive  was assumed to be irreversible in the reverse direction. These criteria form the basis of many existing methods for predicting reaction reversibility [19-21].

However, in our work with the *i*Bsu1103 model we found that  and  alone are insufficient to exhaustively identify every irreversible reaction in a model. Many reactions that are known to be irreversible have a negative  and a positive  due primarily to a lack of knowledge of true metabolite concentration ranges. To identify every irreversible reaction in the *i*Bsu1103 model, we developed and applied a set of three heuristic rules based on common categories of biochemical reactions that are known to be irreversible: carboxylation reactions, phosphorylation reactions, CoA and ACP ligases, ABC transporters, and reactions utilizing ATP hydrolysis to drive an otherwise unfavorable action. We applied our new heuristic rules to identify any irreversible reactions that were missed by previous methods based only on  and .

The first reversibility rule is that all ABC transporters are irreversible. As a result of the application of this rule, ATP synthase is the only transporter in the *i*Bsu1103 model capable of producing ATP directly. The second reversibility rule is that any reaction with a milli-molar Gibbs free energy change (Δ_*r*_*G*^'*m*^) that is less than 2 kcal/mol and greater than -2 kcal/mol is reversible. The Δ_*r*_*G*^'*m *^is calculated by using Equation 6:

(6)

Δ_*r*_*G*^'*m *^is preferred over Δ_*r*_*G*'° when assessing reaction feasibility under physiological conditions because the 1-mM reference state of Δ_*r*_*G*^'*m *^better reflects the intracellular metabolite concentration levels than does the 1-M reference state of Δ_*r*_*G*'°.

The final reversibility rule uses a reversibility score, *S*_*rev*_, calculated as follows:

(7)

In this equation, *n*_*x *_is the number of molecules of type *x *involved in the reaction, *Pi *represents phosphate, *Ppi *represents pyrophosphate, and *λ*_*i *_is a binary parameter equal to 1 when *i *is a low-energy substrate and equal to zero otherwise. Lower-energy substrates in this calculation include CO_2_, HCO_3_^-^, CoA, ACP, phosphate, and pyrophosphate. According to the final reversibility rule, if the product of *S*_*rev *_and Δ_*r*_*G*^'*m *^is >2 and Δ_*r*_*G*^'*m *^is <0, the reaction is irreversible in the forward direction; if the product of *S*_*rev *_and Δ_*r*_*G*^'*m *^is >2 and Δ_*r*_*G*^'*m *^is >0, the reaction is irreversible in the reverse direction. All remaining reactions that fail to meet any of the reversibility rule criteria are considered to be reversible.

### Model optimization procedure overview

We applied an extended version of the GrowMatch procedure developed by Kumar *et al. *[25] to identify changes in the stoichiometry of the *i*Bsu1103 model that would eliminate erroneous model predictions. The procedure consists of four steps applied consecutively (Figure 2): step 1, gap filling to identify and fill gaps in the original model that cause false negative predictions (predictions of zero growth where growth is known to occur); step 2, gap filling reconciliation to combine many gap filling solutions to maximize correction of false negative predictions while minimizing model modifications; step 3, gap generation to identify extra or under-constrained reactions in the gap-filled model that cause false positive predictions (predictions of growth where growth is known not to occur); and step 4, gap generation reconciliation to combine the gap generation solutions to maximize correction of false positive predictions with a minimum of model modifications. While the gap filling and gap generation steps are based entirely on the existing GrowMatch procedure (with some changes to the objective function), the reconciliation steps described here are new.

#### Model optimization step one: gap filling

The gap filling step of the model optimization process, originally proposed by Kumar *et al. *[43], attempts to correct false negative predictions in the original model by either relaxing the reversibility constraints on existing reactions or by adding new reactions to the model. For each simulated experimental condition with a false negative prediction, the following optimization was performed on a superset of reactions consisting of every balanced reaction in the KEGG or in any one of ten published genome-scale models [7,12,20,27,44-49]:

Objective:

(8)

Subject to:

(9)

(10)

(11)

The objective of the gap filling procedure (Equation 8) is to minimize the number of reactions that are not in the original model but must be added in order for biomass to be produced under the simulated experimental conditions. Because the gap filling is run only for conditions with a false negative prediction by the original model, at least one reaction will always need to be added.

In the gap filling formulation, all reactions are treated as reversible, and every reversible reaction is decomposed into separate forward and reverse component reactions. This decomposition of reversible reactions allows for the independent addition of each direction of a reaction by the gap filling, which is necessary for gaps to be filled by the relaxation of the reversibility constraints on existing reactions. As a result of this decomposition, the reactions represented in the gap filling formulation are the forward and backward components of the reactions in the original KEGG/model superset. In the objective of the gap filling formulation, *r*_*gapfilling *_represents the total number of component reactions in the superset; *z*_*i *_is a binary use variable equal to 1 if the flux through component reaction *i *is nonzero; and *λ*_*gapfill*, *i *_is a constant representing the cost associated with the addition of component reaction *i *to the model. If component reaction *i *is already present in the model, *λ*_*gapfill*, *i *_is equal to zero. Otherwise, *λ*_*gapfill*, *i*_is calculated by using Equation 12:

(12)

Each of the *P *variables in Equation 12 is a binary constant representing a type of penalty applied for the addition of various component reactions to the model. These constants are equal to 1 if the penalty applies to a particular reaction and equal to zero otherwise. *P*_*KEGG*, *i *_penalizes the addition of component reactions that are not in the KEGG database. Reactions in the KEGG database are favored because they are up to date and typically do not involve any lumping of metabolites. *P*_*structure*, *i *_penalizes the addition of component reactions that involve metabolites with unknown structures. *P*_*known*-Δ*G*, *i *_penalizes the addition of component reactions for which Δ_*r*_*G*'° cannot be estimated. *P*_*unfavorable*, *i *_penalizes the addition of component reactions operating in an unfavorable direction as predicted by our reaction directionality prediction method. Inclusion of these penalty terms in the *λ*_*gapfill*, *i *_objective coefficients significantly improves the quality of the solutions produced by the gap filling method.

Equation 9 represents the mass balance constraints that enforce the quasi-steady-state assumption of FBA. In this equation, *N*_*super *_is the stoichiometric matrix for the decomposed superset of KEGG/model reactions, and *v *is the vector of fluxes through the forward and reverse components of our superset reactions.

Equation 10 enforces the bounds on the component reaction fluxes (*v*_*i*_), and the values of the component reaction use variables (*z*_*i*_). This equation ensures that each component reaction flux, *v*_*i*_, must be zero unless the use variable associated with the component reaction, *z*_*i*_, is equal to 1. The *v*_*max*, *i *_term in Equation 10 is the key to the simulation of experimental conditions in FBA. If *v*_*max*, *i *_corresponds to a reaction associated with a knocked-out gene in the simulated experiment, this *v*_*max*, *i *_is set to zero. If *v*_*max*, *i *_corresponds to the uptake of a nutrient not found in the media conditions being simulated, this *v*_*max*, *i *_is also set to zero. Equation 11 constrains the flux through the biomass reaction in the model, *v*_*bio*_, to a nonzero value, which is necessary to identify sets of component reactions that must be added to the model in order for growth to be predicted under the conditions being simulated.

Each solution produced by the gap filling optimization defines a list of irreversible reactions within the original model that should be made reversible and a set of reactions not in the original model that should be added in order to fix a single false negative prediction. Recursive mixed integer linear programming (MILP) [50] was applied to identify the multiple gap filling solutions that may exist to correct each false negative prediction. Each solution identified by recursive MILP was implemented in a test model and validated against the complete set of experimental conditions. All incorrect predictions by a test model associated with each gap filling solution were tabulated into an error matrix for use in the next step of the model optimization process: gap filling reconciliation.

#### Model optimization step two: gap filling reconciliation

The gap filling step in the model optimization algorithm produces multiple equally optimal solutions to correct each false negative prediction in the unoptimized model. While all of these solutions repair at least one false negative prediction, they often do so at the cost of introducing new false positive predictions. To identify the cross-section of gap filling solutions that results in an optimal fit to the available experimental data with minimal modifications to the original model, we apply the gap filling reconciliation step of the model optimization procedure. In this step, we perform the following integer optimization that maximizes the correction of false negative errors, minimizes the introduction of new false positive errors, and minimizes the net changes made to the model:

Objective:

(13)

Subject to:

(14)

(15)

(16)

In the objective of the gap filling reconciliation formulation (Equation 13), *n*_*obs *_and *r*_*sol *_are constants representing the total number of experimental observations and the number of unique component reactions involved in the gap filling solutions, respectively; *λ*_*gapfill*, *i *_and *z*_*i *_carry the same definitions as in the gap filling formulation; and *o*_*k *_is a binary variable equal to zero if observation *k *is expected to be correctly predicted given the values of *z*_*i *_and equal to 1 otherwise.

The values of the *o*_*k *_variables are controlled by the constraints defined in Equations 14 and 15. Equation 14 is written for any experimental condition with a false negative prediction by the original model. This constraint states that at least one gap filling solution that corrects this false negative prediction must be implemented in order for this prediction error to be corrected in the gap-filled model. Equation 15 is written for any experimental condition where the original model correctly predicts that zero growth will occur. This constraint states that implementation of any gap filling solution that causes a new false positive prediction for this condition will result in an incorrect prediction by the gap-filled model. In these constraints, *n*_*sol *_is the total number of gap filling solutions; *ε*_*j*, *k *_is a binary constant equal to 1 if condition *k *is correctly predicted by solution *j *and equal to zero otherwise; *s*_*j *_is a binary variable equal to 1 if gap filling solution *j *should be implemented in the gap-filled model and equal to zero otherwise.

The final set of constraints for this formulation (Equation 16) enforce the condition that a gap filling solution (represented by the use variable *s*_*j*_) is not implemented in the gap-filled model unless all of the reaction additions and modifications (represented by the use variable *z*_*i*_) that constitute the solution have been implemented in the model. In these constraints, *γ*_*i*, *j *_is a constant equal to 1 if reaction *i *is involved in solution *j *and equal to zero otherwise.

Once again, recursive MILP was applied to identify multiple equivalently optimal solutions to the gap filling reconciliation problem, and each solution was validated against the complete set of experimental data to ensure that the combination of multiple gap filling solutions did not give rise to additional false positive predictions. The solutions that resulted in the most accurate prediction of growth in all experimental conditions were manually curated to identify the most physiologically relevant solution. This solution was then implemented in the original model to produce the gap-filled model.

#### Model optimization step three: gap generation

The gap-filled model produced by the gap filling reconciliation step not only will retain all of the false positive predictions generated by the original model but also will generate a small number of new false positive predictions that arise as a result of additions and modifications made during the gap filling process. In the gap generation step of the model optimization procedure we attempt to correct these false positive predictions either by removing irreversible reactions or by converting reversible reactions into irreversible reactions. For each simulated experimental condition with a false positive prediction by the gap-filled model, the following optimization was performed:

Objective:

(17)

Subject to:

(18)

(19)

(20)

(21)

(22)

(23)

(24)

(25)

(26)

(27)

The objective of the gap generation procedure (Equation 17) is to minimize the number of component reactions that must be removed from the model in order to eliminate biomass production under conditions where the organism is known not to produce biomass. As in the gap filling optimization, all reversible reactions are decomposed into separate forward and backward component reactions. This process enables the independent removal of each direction of operation of the reactions in the model. As a result, *r*_*gapgen *_in Equation 17 is equal to the number of irreversible reactions plus twice the number of reversible reactions in the gap-filled model; *z*_*i *_is a binary use variable equal to 1 if the flux through component reaction *i *is greater than zero and equal to zero otherwise; *λ*_*gapfill*, *i *_is a constant representing the cost of removal of component reaction *i *from the model. *λ*_*gapfill*, *i *_is calculated using Equation 28:

(28)

The *P*_*irreversible*, *i *_term in Equation 28 is a binary constant equal to 1 if reaction *i *is irreversible and associated with at least one gene in the model. This term exists to penalize the complete removal of reactions from the model (as is done when removing one component of an irreversible reaction) over the adjustment of the reversibility of a reaction in the model (as is done when removing one component of a reversible reaction).

Equations 18 and 19 represent the mass balance constraints and flux bounds that simulate the experimental conditions with false positive predictions. *N*_*gapfilled *_is the stoichiometric matrix for the gap-filled model with the decomposed reversible reactions; *v*_*no*-*growth *_is the vector of fluxes through the reactions under the false positive experimental conditions; and *v*_*max*, *no*-*growth*, *i *_is the upper-bound on the flux through reaction *i *set to simulate the false positive experimental conditions.

Equations 20 and 21 define the dual constraints associated with each flux in the primal FBA formulation. In these constraints, *σ*_*i*, *j *_is the stoichiometric coefficient for metabolite *j *in reaction *i*; *m*_*j *_is the dual variable associated with the mass balance constraint for metabolite *j *in the primal FBA formulation; *μ*_*i *_is the dual variable associated with the upper-bound constraint on the flux through reaction *i *in the primal FBA formulation; and *K *is a large constant selected such that the Equation 20 and 21 constraints are always feasible when *z*_*i *_is equal to zero. Equation 22 sets the dual slack variable associated with reaction *i*, *μ*_*i*_, to zero when the use variable associated with component reaction *i*, *z*_*i*_, is equal to zero. Equation 22 and the term involving *K *in Equations 20 and 21 exist to eliminate all dual constraints and variables associated with component reaction *i *when component reaction *i *is flagged to be removed by the gap generation optimization.

Equation 23 is the constraint that sets the original primal FBA objective (maximization of biomass production) equal to the dual FBA objective (minimization of flux slack). This constraint ensures that every set of *v*_*no*-*growth *_fluxes that satisfies the constraints in Equations 20 to 23 represents an optimal solution to the original FBA problem that maximizes biomass production. Therefore, if the biomass flux is set to zero, as is done in Equation 24, this is equivalent to stating that the binary use variables *z*_*i *_must be set in such a way that the maximum production of biomass when simulating the false positive experimental conditions must be zero.

With no additional constraints, the gap generation optimization would produce solutions recommending the knockout of component reactions that cause the loss of biomass production under every experimental condition instead of just the false positive conditions. Constraints are required to ensure that only solutions that eliminate biomass production under the false positive conditions while preserving biomass production in all other conditions will be feasible. These constraints are defined by Equations 25, 26, and 27, which represent the FBA constraints simulating an experimental condition where the organism being modeled is known to grow. When the false positive condition being simulated by the *v*_*max*, *no*-*growth*, *i *_values is the knockout of an essential gene or interval, the *v*_*max*, *growth*, *i *_values in Equation 26 simulate the same media conditions with no reactions knocked out. When the false positive condition being simulated is an unviable media, the *v*_*max*, *growth*, *i *_values simulate a viable media. Because the binary *z*_*i *_variables are shared by the 'no growth' and 'growth' FBA constraints, *z*_*i *_will be set to zero only for those reactions that are not essential or coessential under the 'growth' conditions but are essential or coessential under the 'no growth conditions'. To further reduce the probability that a gap generation solution will cause new false negative predictions, we identified the component reactions in the gap-filled model that were essential for the correct prediction of growth in at least three of the experimental conditions prior to running the gap generation optimization. The *z*_*i *_variables associated with these essential component reactions were fixed at one to prevent their removal in the gap generation optimization.

As done in previous steps, recursive MILP was used to identify up to ten equally optimal solutions that correct each false positive prediction error in the gap-filled model. Each solution was implemented and validated against the complete set of experimental data, and the accuracy of each solution was tabulated into a matrix for use in the final step of the model optimization procedure: gap generation reconciliation.

#### Model optimization step four: gap generation reconciliation

Like the gap filling step, the gap generation step of the model optimization process produces multiple equally optimal solutions to correct each false positive prediction in the gap-filled model, and many of these solutions introduce new false negative prediction errors. To identify the cross-section of gap generation solutions that results in the maximum correction of false positive predictions with the minimum addition of false negative predictions, we perform one final optimization step: gap generation reconciliation. The optimization problem solved in the gap generation reconciliation step is identical to the gap filling reconciliation optimization except that the constraints defined by Equations 14 and 15 are replaced by the constraints defined by Equations 29 and 30:

(29)

(30)

Equation 29 is written for any experimental condition with a false positive prediction by the gap-filled model. This constraint states that at least one gap generation solution that corrects the false positive prediction must be implemented for the condition to be correctly predicted by the optimized model. Equation 30 is written for any experimental condition where the original model correctly predicts that growth will occur. This constraint states that implementation of any gap generation solution that causes a new false positive prediction will result in a new incorrect prediction by the optimized model. All of the variables and constants used in Equations 29 and 30 have the same meaning as in Equations 14 and 15.

Although the objective, remaining constraints, and remaining variables in the gap generation reconciliation are mathematically identical to the gap filling reconciliation, some variables take on a different physiological meaning. Because gap generation solutions involve the removal (not the addition) of reactions from the gap-filled model, the reaction use variable *z*_*i *_is now equal to 1 if a reaction is to be removed from the gap-filled model and equal to zero otherwise.

The gap generation reconciliation was solved repeatedly by using recursive MILP to identify multiple solutions to the gap generation reconciliation optimization, and each solution was implemented in a test model and validated against the complete set of experimental data. The solutions associated with the most accurate test models were manually examined to identify the most physiologically relevant solution. The selected solution was then implemented in the gap-filled model to produce the optimized *i*Bsu1103 model.

## Abbreviations

Δ_*f*_*G*'°: standard Gibbs free energy change of formation; Δ_*r*_*G*'°: standard Gibbs free energy change of reaction; Δ_*r*_*G*^'*m*^: milli-molar Gibbs free energy change of reaction; : maximum Gibbs free energy change of reaction; : minimum Gibbs free energy change of reaction; ACP: acyl-carrier-protein; BOF: biomass objective function; CoA: coenzyme A; CDW: cell dry weight; FBA: flux balance analysis; KEGG: Kyoto Encyclopaedia of Genes and Genomes; LB: Luria-Bertani; MILP: mixed integer linear programming.

## Authors' contributions

CH, JZ, MC, and RS all participated in the reconstruction, curation, and analysis of the *i*Bsu1103 model. CH developed the reaction reversibility prediction and model optimization methods with direction and advice provided by RS. RS conceived of the project and coordinated all research. CH wrote the paper with revisions by JZ, MC, and RS. All authors read and approved the final manuscript.

## Additional data files

The following additional data are available with the online version of this paper: an Excel file containing all supplementary data associated with the *i*Bsu1103 model as Tables S1 to S11 (Additional data file 1); a zip archive containing tab-delimited text files for all of the supplementary tables included in Additional data file 1 (Additional data file 2); a zip archive containing data on the structure of every molecule in the model in molfile format (Additional data file 3); an SBML version of the model, which may be used with the published COBRA toolbox [18] to run FBA on the model (Additional data file 4).

## Supplementary Material

Additional data file 1 Tables S1 and S2 contain all compound and reaction data associated with the model, respectively; Table S3 lists all of the open problem reactions in the model; Table S4 lists all of the essential genes that have nonessential homologs in the *B. subtilis *genome; Table S5 lists all of the changes made to the model during the model optimization process; Table S6 lists the reactions in the Oh *et al. *model that are not in the *i*Bsu1103 model; Table S7 shows simulation results for all 1,500 experimental conditions; Table S8 provides the details on the media formulations used for each FBA simulation; and Tables S9, S10, and S11 show all data on the genes, functional roles, and subsystems in the *B. subtilis *SEED annotation. Click here for file

Additional data file 2 The text files may be copied into any spreadsheet program of choice to visualize the data for the *i*Bsu1103 model. Click here for file

Additional data file 3 These molfiles reflect the structure of the predominant ionic form of the compounds at neutral pH as predicted using the MarvinBeans software [28]. These structures were used with the group contribution method [40-42] to estimate the Δ_*f*_*G*'° and Δ_*r*_*G*'° for the compounds and reactions in the model. Click here for file

Additional data file 4 This may be used with the published COBRA toolbox [18] to run FBA on the model. Click here for file
